# Evaluation of reference genes for gene expression in red-tailed phascogale (*Phascogale calura*) liver, lung, small intestine and spleen

**DOI:** 10.7717/peerj.2552

**Published:** 2016-10-13

**Authors:** Oselyne T.W. Ong, Lauren J. Young, Julie M. Old

**Affiliations:** School of Science and Health, Western Sydney University, Richmond, New South Wales, Australia

**Keywords:** Immunity, Reference genes, Real-time PCR, Marsupial, qPCR, Housekeeping genes

## Abstract

**Background:**

Reference genes serve an important role as an endogenous control/standard for data normalisation in gene expression studies. Although reference genes have recently been suggested for marsupials, independent analysis of reference genes on different immune tissues is yet to be tested. Therefore, an assessment of reference genes is needed for the selection of stable, expressed genes across different marsupial tissues.

**Methods:**

The study was conducted on red-tailed phascogales (*Phascogale calura*) using five juvenile and five adult males. The stability of five reference genes (glyceraldehyde-3-phosphate dehydrogenase, *GAPDH*; *β*-actin, *ACTB*; *18S* rRNA, *18S; 28S* rRNA*, 28S;* and ribosomal protein L13A, *RPL13A*) was investigated using SYBR Green and analysed with the geNorm application available in qBase^PLUS^ software.

**Results:**

Gene stability for juvenile and adult tissue samples combined show that *GAPDH* was most stable in liver and lung tissue, and *18S* in small intestine and spleen. While all reference genes were suitable for small intestine and spleen tissues, all reference genes except *28S* were stable for lung and only *18S* and *28S* were stable for liver tissue. Separating the two age groups, we found that two different reference genes were considered stable in juveniles (*ACTB* and *GAPDH*) and adults (*18S* and *28S*), and *RPL13A* was not stable for juvenile small intestine tissue. Except for *28S*, all reference genes were stable in juvenile and adult lungs, and all five reference genes were stable in spleen tissue.

**Discussion:**

Based on expression stability, *ACTB* and *GAPDH* are suitable for all tissues when studying the expression of marsupials in two age groups, except for adult liver tissues. The expression stability between juvenile and adult liver tissue was most unstable, as the stable reference genes for juveniles and adults were different. Juvenile and adult lung, small intestine and spleen share similar stable reference genes, except for small intestine tissues where all reference genes were stable in adults but *RPL13A* was not suitable in juveniles.

## Introduction

Fluorescence-based quantitative real-time polymerase chain reaction (qPCR) has the capacity to monitor the amplification of cDNA during thermocycling, starting with the use of ethidium bromide for the detection of fluorescence (Higuchi et al., 1992). Over the past 13 years, qPCR has developed into the most accurate and sensitive method to study gene expression with low concentrations of mRNA (Bustin, 2000; Schmittgen & Livak, 2008). When studying the expression of a target gene, it is important to have a stable reference gene for the normalisation of gene expression (Pierzchała et al., 2011). Although it may seem scientifically sound to use reference genes that have been used in other similar studies, it has to be reported that common reference genes such as *GAPDH* and *ACTB* have various stabilities depending on the type of tissue or experimental conditions (Glare et al., 2002; Selvey et al., 2001; Zhong & Simons, 1999). Commonly used rRNA genes, 18S and 28S are also affected by various biological factors (Warner, 1999). At present, there has not been a study on the stability of reference genes in a marsupial. In most cases, reference genes found stable in other mammalian groups (particularly eutherians) have been used in studies without considering the stability of the reference genes, which may differ between mammal groups and tissues or cells.

Reference genes should not only be stable enough for normalisation, but the use of more than one reference gene is encouraged in expression studies as the use of only one reference gene has led to expression study errors (Bustin, 2000; Vandesompele et al., 2002). By selecting the most stable reference genes for the type of tissue/cells or experimental condition, an accurate normalisation of qPCR can be performed, avoiding variation in reference gene expression in tissues/cells investigated (Vandesompele et al., 2002). Since the stability of reference genes have not been reported in a marsupial, gene expression studies using similar tissues/cells or marsupial species can use this study as a baseline, and therefore be used in future marsupial gene expression studies.

The aim of this study was to identify stable reference genes across a range of tissues of a marsupial species—the Red-tailed phascogale (*Phascogale calura*). Red-tailed phascogales are a model species as they are small and relatively easy to maintain in captivity (Foster et al., 2006; Russell, 1982; Stannard et al., 2013). In the wild, the red-tailed phascogale inhabits a small corner of the south west of western Australia (Bradley, Foster & Taggart, 2008) and are distinguished from other small marsupials by their long brush-like hairs on the end of their red tail (Kennedy & Williams, 1990).

This study evaluates the expression stability of five different reference genes in four different tissues associated with immunity in the red-tailed phascogale; liver, lung, small intestine and spleen in two different age groups. Optimal reference genes should be considered stable and expressed at constant levels in various tissues and age groups. Liver and lung tissues were primarily chosen because they contain large populations of macrophages and are in regular contact with pathogens (Laskin, Weinberger & Laskin, 2001). In addition, unlike eutherian livers that cease haematopoiesis prior to birth, the marsupial liver is the main site of haematopoiesis during early postnatal life (reviewed by Borthwick, Young & Old (2014); Old & Deane (2000)).

Lung and small intestine were chosen as representative mucosal-associated lymphoid tissue (MALT). The MALT in lungs is responsible for protection of the respiratory system (Mak, Saunders & Jett, 2013), whilst the small intestine is an important gut-associated lymphoid tissue (GALT). In marsupials, GALT can be localised in Peyer’s patches or follicular aggregations, or appear as scattered cells distributed throughout the gut (Old & Deane, 2002). The last of the tissues chosen for use in this study was spleen. The spleen is an important haematopoietic site and actively involved in the adaptive immune response (reviewed in Borthwick, Young & Old (2014); Old & Deane (2000)). The four red-tailed phascogale tissues (liver, lung, small intestine and spleen) were therefore chosen based on their immunological capacity and function. The expression stability of five reference genes in these tissues was investigated using the geNorm application in the qBase^PLUS^ software.

## Materials and Methods

### Animal and tissue collection

Ten male red-tailed phascogales from two age groups (juveniles: 3.5–5 months and adults: 1.2–1.5 years) were utilised in this study. Tissue samples were opportunistically obtained from a the Small Native Mammal Teaching and Research Facility, a captive colony housed at the Western Sydney University (WSU) (Richmond, NSW) as per standard operating procedures approved by the UWS Animal Ethics Committee (A9694) during population maintenance Samples of liver, lung, small intestine and spleen were dissected, and immediately stored at −80 °C until total RNA extraction.

### RNA extraction and cDNA synthesis

Total RNA was extracted using the SV Total RNA Isolation System (Promega, Wisconsin, USA) according to the manufacturer’s protocol. The quantity and quality of total RNA was estimated using a Nanodrop 2000 Spectrophotometer (Thermo Scientific, Wilmington, Delaware, USA) with the OD260 nm/OD280 nm ratio expected to be between 1.8 and 2. One µg of total RNA was reverse transcribed with SuperScript^®^ III First-Strand Synthesis SuperMix (Invitrogen, Carlsbad, California, USA) according to the manufacturer’s protocol. The quantity of the final cDNA was assessed using a Nanodrop 2000 Spectrophotometer, and final cDNA products were aliquoted and stored at −20 °C until use.

### Primers and real-time PCR

We selected five reference genes used previously in marsupial gene expression studies (Maher et al., 2014; Markey et al., 2007; Yu et al., 2006): glyceraldehyde-3-phosphate dehydrogenase (*GAPDH*), *β*-actin (*ACTB*), 18*S* rRNA (18*S*), 28*S* rRNA (28*S*) and ribosomal protein L13a (*RPL13A*). Real-time PCR primers, *GAPDH* and *ACTB*, were designed using consensus sequences based on marsupial species obtained from GenBank (Tasmanian devil *Sarcophilus harrisii*) *GAPDH*: XM_012550750.1; gray short-tailed opossum (*Monodelphis domestica*) *GAPDH*: XM_007503905.1; Tasmanian devil *ACTB*: XM_003761554.2). Primers for 18*S* and 28*S* were obtained from Daly et al. (2009), and *RPL13A* from Siddle et al. (2013). Specific information for each primer is listed in Table 1.

**Table 1:** Candidate reference genes evaluated in this study.

Gene symbol	Gene name	Oligo sequence (5′→ 3′)	Amplicon size (bp)	Annealing temp. (°C)
*GAPDH*	Glyceraldehyde-3-phosphate dehydrogenase	Forward: CAGGCGGAGTAGACATTG	63	60
		Reverse: CCTTGAACTTGCCATGGG		
*ACTB*	*β*-actin	Forward: TTGCTGACAGGATGCAGAAG	66	60
		Reverse: GAGCCTCCAATCCAGACAGA		
		Rindfleisch et al. (2010)		
28*S*	28*S* Ribosomal RNA	Forward: CGATGTCGGCTCTTCCTATC	165	60
		Reverse: TCCTCAGCCAAGCACATACA*		
		Daly et al. (2009)		
		*Reverse primer was modified according to marsupial sequences.		
18*S*	18*S* Ribosomal RNA	Forward: CCAACACGGGAAACCTCA	83	60
		Reverse: AACCAGAAATCGCTCCAC		
		(Daly et al., 2009)		
*RPL13A*	Ribosomal protein L13a	Forward: CCCCACAAGACCAAGCGAGGC	146	60
		Reverse: ACAGCCTGGTATTTCCAGCCAACC		
		(Siddle et al., 2013)		

PCR amplification was performed using a Rotor Gene Q (Qiagen, Hilden, Germany) and the Rotor-Gene SYBR^®^ Green PCR kit (Qiagen). A PCR mix (25 µL) was prepared: 7.5 µL water, 2.5 µL primers (forward and reverse; 10 µM), 1 µL (100 ng) cDNA, and 12.5 µL of Rotor-Gene SYBR Green PCR Master Mix. The following amplification program was used: 5 min denaturation at 95 °C, 35 cycles of amplification with 5 s at 95 °C (denaturation), 10 s at 60 °C (annealing), and 15 s at 72 °C (elongation). Annealing temperatures were optimised according to individual genes and primers by testing several annealing temperatures ranging from 55 °C to 65 °C around the respective primer Tm, and the annealing temperature with the best efficiency was chosen. A melting step was performed to confirm a single gene-specific peak by a stepwise temperature increase ranging from 60 °C to 95 °C at ramp rate 1 °C/s with continuous monitoring of fluorescence. Further analysis of amplicon specificity and size were also evaluated running qPCR products in a standard 2% agarose gel electrophoresis. Standard curves were made to calculate the amplification efficiency during real-time PCR using five-fold serial dilutions of cDNA for each tissue and each reference gene in one adult male red-tailed phascogale. The quantification cycle (*C*_*q*_) was automatically determined for each reaction by the Rotor Gene Software (v. 1.7.94)

### Data analysis

Gene expression variation was calculated for individual reference genes based on cycle threshold (*C*_*q*_) values and real-time PCR efficiencies (*E*). The real-time *E* value was calculated from the given slopes in the qBase^PLUS^ software (Hellemans et al., 2007) according to the equation: (*E* = 10(−1∕slope) − 1). Only *C*_*q*_ values <40 were used for calculation of *E* values. *C*_*q*_ and *E* values were then analysed in geNorm on the qBase^PLUS^ software, which ranks the reference genes based on the *M* values (reference genes with the lowest *M* value is considered most stable). A one-way ANOVA was also performed on *C*_*q*_ values obtained from the expression in juveniles and adults of the four reference genes.

## Results

**Figure 1:**
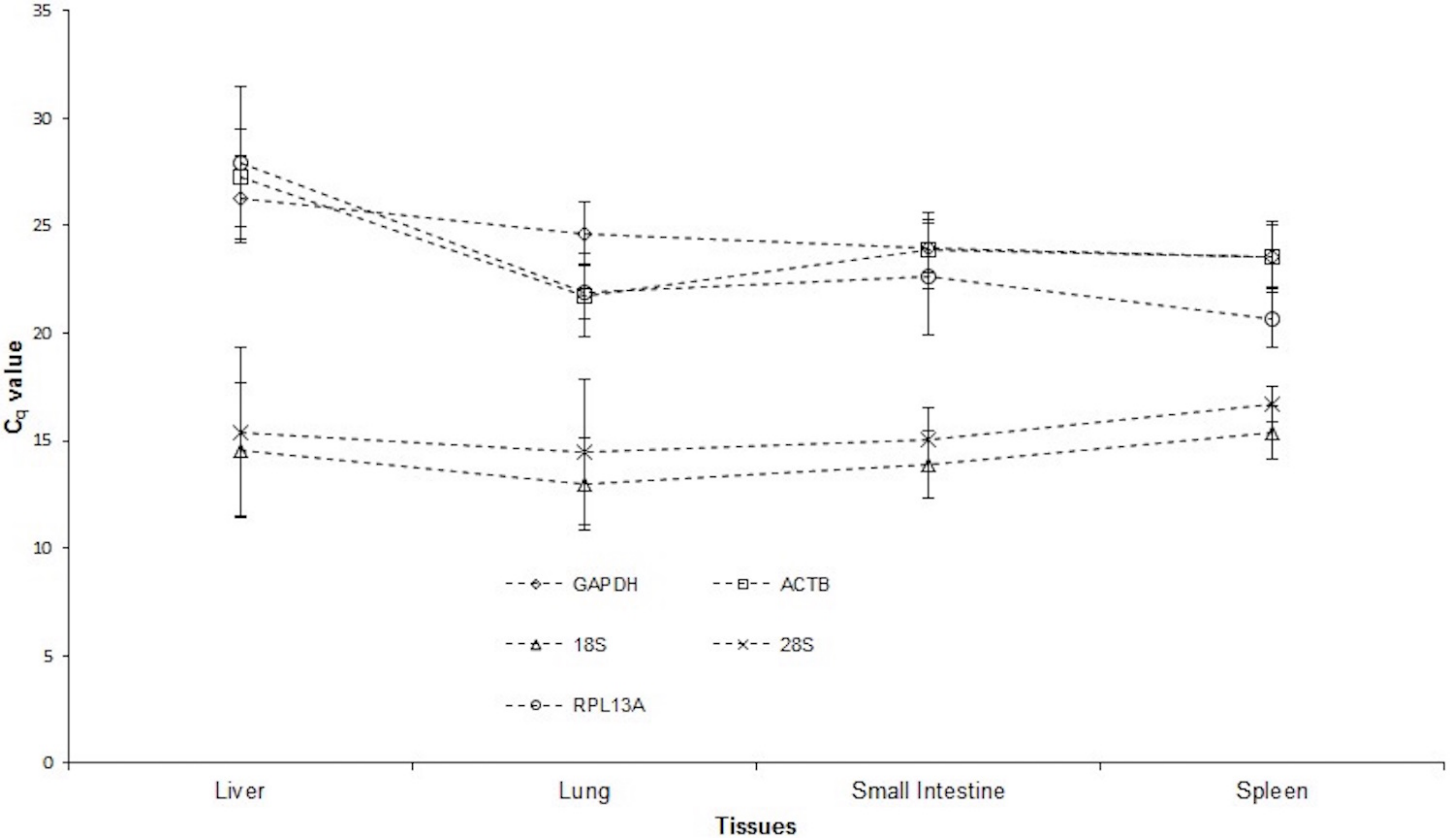
Average RNA transcription levels of putative reference genes presented as absolute *C*_*q*_ values. The reference genes are: Glyceraldehyde 3-phosphate dehydrogenase (*GAPDH*), *β*-actin (*ACTB*), 18S rRNA (*18S*), 28S rRNA (*28S*) and ribosomal protein L13a (*RPL13A*) in liver, lung, small intestine and spleen tissue samples.

Five reference genes were amplified in four tissues, and all real-time PCR assays produced a single peak on the melting curve (*GAPDH*, Fig. S1; *ACTB*, Fig. S2; 18*S*, Fig. S3; 28*S*, Fig. S4 and *RPL13A*, Fig. S5) and have been submitted into GenBank. GenBank accession numbers are as follows: *GAPDH,* KX788916; *ACTB*, KX78891; *18S*, KX788914; *28S*, KX788915 and *RPL13*A, KX788918. The linear correlation coefficient (R^2^) of all genes ranged from 0.97–1.00. *C*_*q*_ values for all genes in all samples were within 10.41–33.19 cycles, and were covered by the range of their respective standard curves. *E* value of reference genes, mean *C*_*q*_ values and range of *C*_*q*_ values for each tissue are depicted in Table 2. All reference genes in all tissues had a *C*_*q*_value below 29, indicating an abundance of target nucleic acid in cDNA samples (Fig. 1). *C*_*q*_ values observed for all reference genes in this study were insignificant in juvenile and adult tissues (*p* ≥ 0.05).

**Table 2:** Cycle threshold (*C*_*q*_) and reaction efficiency (*E*) values for individual genes in examined tissues of juveniles and adults.

	*GAPDH*	*ACTB*	*18S*	*28S*	*RPL13A*
**Liver**					
Mean *C*_*q*_	26.242	27.226	14.526	15.382	27.907
Range of *C*_*q*_	22.55–29.98	22.07–30.09	9.66–18.48	9.83–21.28	22.27–33.19
*E* (out of 2)	1.855 ± 0.047	1.716 ± 0.08	1.953 ± 0.035	1.89 ± 0.011	1.851 ± 0.029
**Lung**					
Mean *C*_*q*_	24.6	21.746	12.995	14.476	21.924
Range of *C*_*q*_	22.74–28.06	19.17–24.84	10.41–16.64	10.8–20.14	19.72–24.43
*E* (out of 2)	1.94 ± 0.058	1.732 ± 0.04	1.912 ± 0.026	1.909 ± 0.035	1.763 ± 0.032
**Small intestine**					
Mean *C*_*q*_	23.981	23.849	13.867	15.04	22.618
Range of *C*_*q*_	21.75–25.50	20.67–27.47	11.56–17.67	12.32–17.50	18.89–28.53
*E*(out of 2)	1.919 ± 0.043	1.753 ± 0.058	1.833 ± 0.033	1.703 ± 0.013	1.811 ± 0.067
**Spleen**					
Mean *C*_*q*_	23.541	23.566	15.361	16.673	20.661
Range of *C*_*q*_	21.24–26.89	21.64–26.56	13.46–17.81	14.8–17.59	18.92–23.40
*E*(out of 2)	1.879 ± 0.033	1.693 ± 0.043	1.768 ± 0.036	1.978 ± 0.029	1.801 ± 0.028

**Figure 2:**
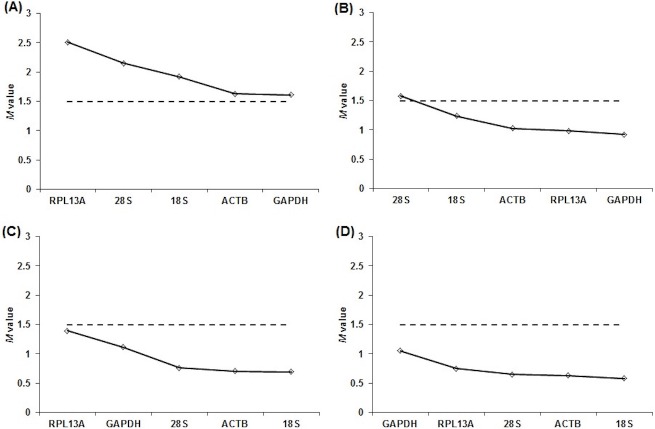
Overall expression stability values (*M*) of reference genes in tissues of different ages. Gene expression stability of reference genes in juvenile and adult red-tailed phascogale tissues (A) liver, (B) lung, (C) small intestine and (D) spleen analysed by geNorm application (qBase^PLUS^). The reference genes are: Glyceraldehyde 3-phosphate dehydrogenase (*GAPDH*), *β*-actin (*ACTB*), 18S rRNA (*18S*), 28S rRNA (*28S*) and ribosomal protein L13a (*RPL13A)*. Genes with the lowest *M* values have the most stable expression.

*C*_*q*_ and *E* values were used in qBase^*PLUS*^ to calculate expression stability (*M* value). *M* values are used to rank reference genes based on the stability, depending on the type of tissue (Fig. 2). *M* values below 1.5 indicate a stable expression (Nygard et al., 2007). According to the results obtained, the most stable genes in liver are *ACTB* and *GAPDH* lungs *RPL13A* and *GAPDH* small intestine *ACTB* and 18*S* and spleen *ACTB* and 18*S*.

As per Nygard et al. (2007), *M* values lower than 1.5 were considered stable reference genes. Upon analysis using the geNorm application, *M* values for *ACTB* and *GAPDH* in all liver tissue samples were below 1.5, indicating their stability for use in the developing red-tailed phascogale studies. In addition, all reference genes except 28*S* were stable for lung tissue, and all reference genes were stable in both small intestine and spleen tissue samples (Fig. 2 and Table 3).

**Figure 3:**
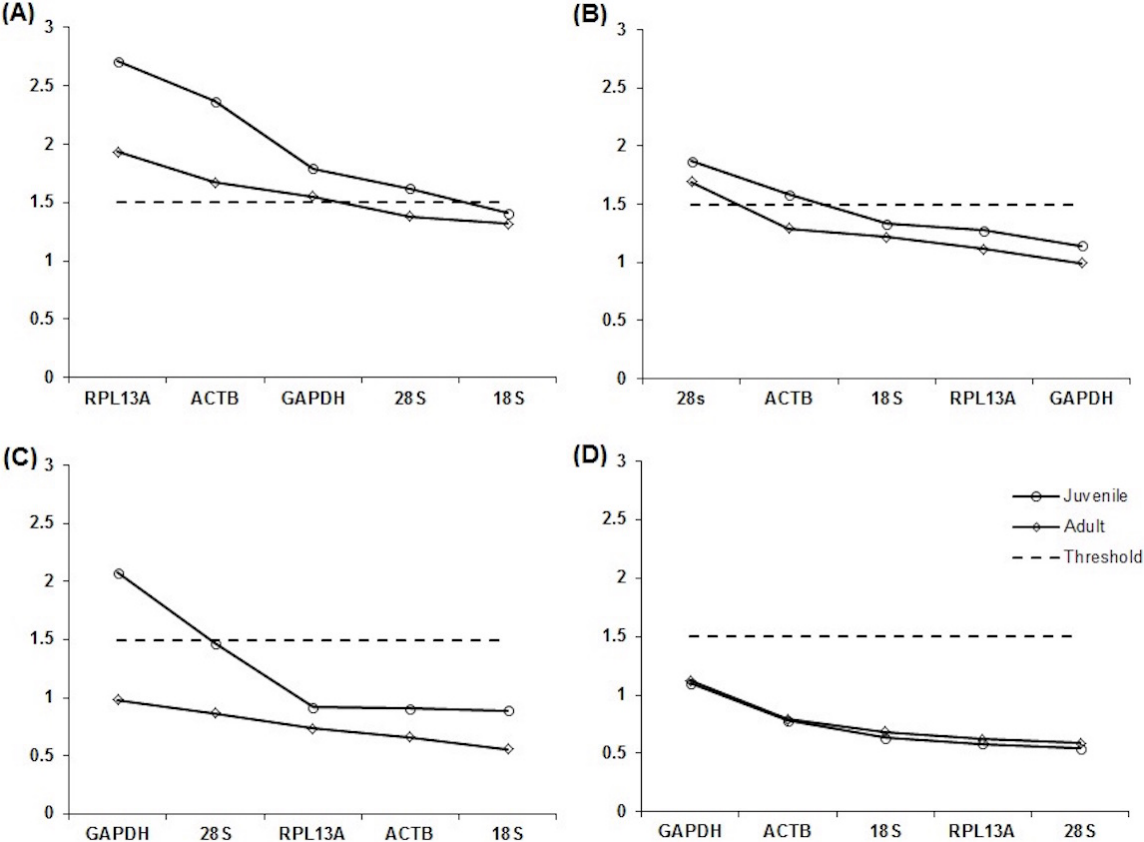
Expression stability values (*M*) of reference genes in tissues of juvenile and adults. Gene expression stability of reference genes in juvenile and red-tailed phascogale tissues (A) liver, (B) lung, (C) small intestine, (D) spleen, analysed by geNorm application (qBase^PLUS^). The reference genes are: Glyceraldehyde 3-phosphate dehydrogenase (*GAPDH*), *β*-actin (*ACTB*), 18S rRNA (*18S*), 28S rRNA (*28S*) and ribosomal protein L13a (*RPL13A)*. Genes with the lowest *M* values have the most stable expression.

When the two age groups were analysed individually, all reference genes had *M* values below 1.5 for adult small intestine, juvenile and adult spleen tissue samples. The two most stable reference genes differed in juvenile and adult liver tissues: *M* values for 18*S* and 28*S* for adult liver samples were considered stable, whereas *ACTB* and *GAPDH* were stable in juvenile liver samples. All reference genes except 28*S* were considered stable in both juvenile and adult lung samples, and all reference genes except *RPL13A* were considered stable in juvenile small intestine tissue samples (Fig. 3).

## Discussion

The ideal reference gene should constantly be transcribed in the type of tissue being examined (Nygard et al., 2007). Studies looking at the expression of reference genes in multiple tissues have however demonstrated that the regulation of reference genes are tissue specific (Lisowski et al., 2008). In this study, we provided a detailed analysis of the stability and expression levels of five different reference genes previously used in marsupial expression studies (Daly et al., 2009; Maher et al., 2014; Menzies et al., 2009; Siddle et al., 2013), in four different red-tailed phascogale tissues. We found at least two stable reference genes with *M* values >1.5 for liver, lung, small intestine and spleen, and that all reference genes were suitable for expression studies of red-tailed phascogale small intestine and spleen tissues, which is useful as the normalisation of gene expression requires at least two reference genes (Bustin, 2000). The average *C*_*q*_ values for all reference genes in all tissues were below 29 cycles, indicating strong positive reactions of the target tissue to the reference genes (Fig. 1).

**Table 3:** Control genes ranked in order of their expression stability^a^. Reference genes with *M* values below 1.5 are considered as suitable reference genes for a particular immune tissue and are bold.

Liver	Lung	Small intestine	Spleen
*RPL13A*	28*S*	***RPL13A***	***GAPDH***
28*S*	***18S***	***GAPDH***	***RPL13A***
18*S*	***ACTB***	***28S***	***28S***
***ACTB***	***RPL13A***	***ACTB***	***ACTB***
***GAPDH***	***GAPDH***	***18S***	***18S***

**Notes.**

a Increasing expression stability from top to bottom.

Not unexpectedly, the results of this study showed that expression stability differs between different tissues, and confirm that reference genes are expressed in every cell but are regulated differently in different tissues (Lisowski et al., 2008). *GAPDH* is one of the most commonly used reference genes for normalisation in mammalian tissues. Studies have found *GAPDH* expression to be unstable as its expression differs, for example, according to age and sex of individuals (Barber et al., 2005). Therefore, it was not surprising that there were significant differences in *GAPDH* expression across tissues used in this study; *GAPDH* expression was most stable for liver and lung, and least stable for spleen tissues (Fig. 2 and Table 3) When separated into two age groups, *GAPDH* expression had the highest stability in juvenile liver tissues, and a combination of the two ages. *GAPDH*, along with *ACTB*, were stable for all tissue samples, except for adult liver tissues.

*ACTB* is another reference gene commonly used for normalisation in mammalian tissues (Menzies et al., 2012; Nygard et al., 2007). Foss, Baarsch & Murtaugh (1998) found that the levels of *ACTB* were more variable than GAPDH, and that high levels of *ACTB* were found in the porcine small intestine and spleen. However, we found that *ACTB* was more stable than *GAPDH* in small intestine and spleen tissue. In particular, juvenile small intestine and spleen tissues had the highest *ACTB* stability. Selvey et al. (2001) found that *ACTB* is an unstable reference gene in mouse sarcoma cells (matrigel), and found 18*S* to be more stable. When combining both age groups, our study agrees with Selvey et al. (2001), as *ACTB* was suitable for normalisation in small intestine and spleen tissue, however it was still less stable than 18*S* in the same tissues. The same results were also observed using adult small intestine and spleen tissue samples.

Both 18*S* and 28*S* are often recommended as reference genes because ribosomal RNA has little variation among mammalian tissues and is often used as a successful internal standard (Goidin et al., 2001; Thellin et al., 1999). In addition, 18*S* and 28*S* were found to work effectively for normalisation in marsupial tissues (Janke et al., 2002; Maher et al., 2014). In this study 18*S* and 28*S* were suitable for normalisation in small intestine and spleen tissue, with 18*S* being the most stable. 18*S* and 28*S* were also the only two stable reference genes for adult liver tissue.

The last reference gene used in this study was *RPL13A*, a gene that encodes a protein in the 60*S* subunit of ribosomes (Vandesompele et al., 2002). Szabo et al. (2004) found *RPL13A* to be the best universal reference gene in various human tissues, including lung and small intestine. In addition, *RPL13A* had been selected by Ahn et al. (2008) as one of two ideal reference genes in rhesus macaques (*Macaca mulatta*), which included the comparison of eight reference genes in six tissues, including liver and lung. In this study, *RPL13A* was found to be least stable (highest *M*value) in liver and small intestine tissue, but suitable for normalisation in lung, small intestine and spleen tissue when both ages are combined. When divided into different age groups, *RPL13A* was stable for both age groups for lung and spleen tissue and adult small intestine, showing its suitability for normalisation in most tissue samples, with the exception of liver.

The findings of this study confirmed previous research that demonstrated tissue specific regulation of some reference genes in eutherian mammals (Lisowski et al., 2008; Nygard et al., 2007; Uddin et al., 2011) also apply to a marsupial. Pierzchala et al. (2011) and Uddin et al. (2011) did not identify any of the reference genes used in this study, as a stable reference gene in porcine liver, which shows that the regulation of certain reference genes may be different in marsupials. This study also demonstrated the stability of reference genes in some, but not all, marsupial tissues that were tested. This will aid in the selection of reference genes for normalisation in future expression studies in marsupials, particularly where studies of immune-related whole tissue preparations are performed. As in eutherian mammals, marsupial expression studies are increasing because of the ability of qPCR to detect and quantify nucleic acids (Bustin, 2000). For example, expression studies have been conducted in koala (*Phascolarctos cinereus*) to test the up- or down-regulation of specific immune genes in stimulated cells (Maher et al., 2014), and whether viral RNA levels increased or decreased in association with age (Tarlinton et al., 2005). Expression studies are also useful for comparing gene expression in eutherians and marsupials (Hübler et al., 2013).

## Conclusions

We have successfully found stable reference genes in lung, small intestine and spleen tissue preparations from a dasyurid marsupial. It is possible to apply this study to whole tissue gene expression studies, especially when it is associated with immunity. While gene expression may occur at the single-cell level, whole tissue studies show the mean expression of several cell types available in the tissue (Kahlem et al., 2004), which is relevant especially when more than one type of cell relates to immunity in an immune system study. Future studies that focus on isolated cell preparations from these tissues will shed further light on reference gene expression and whether or not whole tissue preparations can be directly compared with cell culture studies. Results from the present study enable recommendations on reference genes suitable for use in various marsupial tissues and for normalisation in gene expression experiments in developing marsupials.

##  Supplemental Information

10.7717/peerj.2552/supp-1 Figure S1 Melting curves for glyceraldehyde 3-phosphate dehydrogenase (*GAPDH*) expression The melting curves for *GAPDH* expression in (A) liver, (B) lung, (C) small intestine and (D) spleen of red-tailed phascogales (*n* = 10), performed in triplicate.

10.7717/peerj.2552/supp-2 Figure S2 Melting curves for *β*-actin (*ACTB*) expression The melting curves for *ACTB* expression in (A) liver, (B) lung, (C) small intestine and (D) spleen of red-tailed phascogales (*n* = 10, performed in triplicate.

10.7717/peerj.2552/supp-3 Figure S3 Melting curves for 18S rRNA (*18S*) The melting curves for *18S* expression in (A) liver, (B) lung, (C) small intestine and (D) spleen of red-tailed phascogales (*n* = 10), performed in triplicate.

10.7717/peerj.2552/supp-4 Figure S4 Melting curves for 28S rRNA (*28S*) The melting curves for *28S* expression in (A) liver, (B) lung, (C) small intestine and (D) spleen of red-tailed phascogales (*n* = 10), performed in triplicate

10.7717/peerj.2552/supp-5 Figure S5 Melting curves for ribosomal protein L13a (*RPL13A*) The melting curves for *RPL13A* expression in (A) liver, (B) lung, (C) small intestine and (D) spleen of red-tailed phascogales (*n* = 10), performed in triplicate.

10.7717/peerj.2552/supp-6 Supplemental Information 1 Glyceraldehyde 3-phosphate dehydrogenase (GAPDH) raw data by the Rotor Gene machine showing the details of the CT values obtained

10.7717/peerj.2552/supp-7 Supplemental Information 2 Actin Beta (ACTB) raw data by the Rotor Gene machine showing the details of the CT values obtained

10.7717/peerj.2552/supp-8 Supplemental Information 3 18s rRNA raw data by the Rotor Gene machine showing the details of the CT values obtained

10.7717/peerj.2552/supp-9 Supplemental Information 4 28s rRNA raw data by the Rotor Gene machine showing the details of the CT values obtained

10.7717/peerj.2552/supp-10 Supplemental Information 5 Ribosomal protein L13A (RPL13A) by the Rotor Gene machine showing the details of the CT values obtained

## List of abbreviations

GAPDH: Glyceraldehyde-3-phosphate dehydrogenase
ACTB: *β*-actin
28S: 28*S* Ribosomal RNA
18S: 18*S* Ribosomal protein L13a
Cq: Cycle Threshold
E: Reaction efficiency
M: Expression stability value
qPCR: Real-time polymerase chain reaction
MALT: Mucosal-associated lymphoid tissue
GALT: Gut-associated lymphoid tissue
WSU: Western Sydney University
PCR: Polymerase chain reaction

## Additional Information and Declarations

### DNA Deposition

The following information was supplied regarding the deposition of DNA sequences: The following are sequences that have been submitted into GenBank. GAPDH: KX788916
ACTB: KX788917
18s rRNA: KX788914
28s rRNA: KX788915
RPL13a: KX788918

### Data Availability

The following information was supplied regarding data availability: The raw data has been supplied as Supplemental Information.
