# Supplementary figures and images for: Evaluation of reference genes for gene expression in red-tailed phascogale (Phascogale calura) liver, lung, small intestine and spleen
Source: PeerJ. 2016 Oct 13;4:e2552. doi: 10.7717/peerj.2552 (PMC5068414; doi:10.7717/peerj.2552)

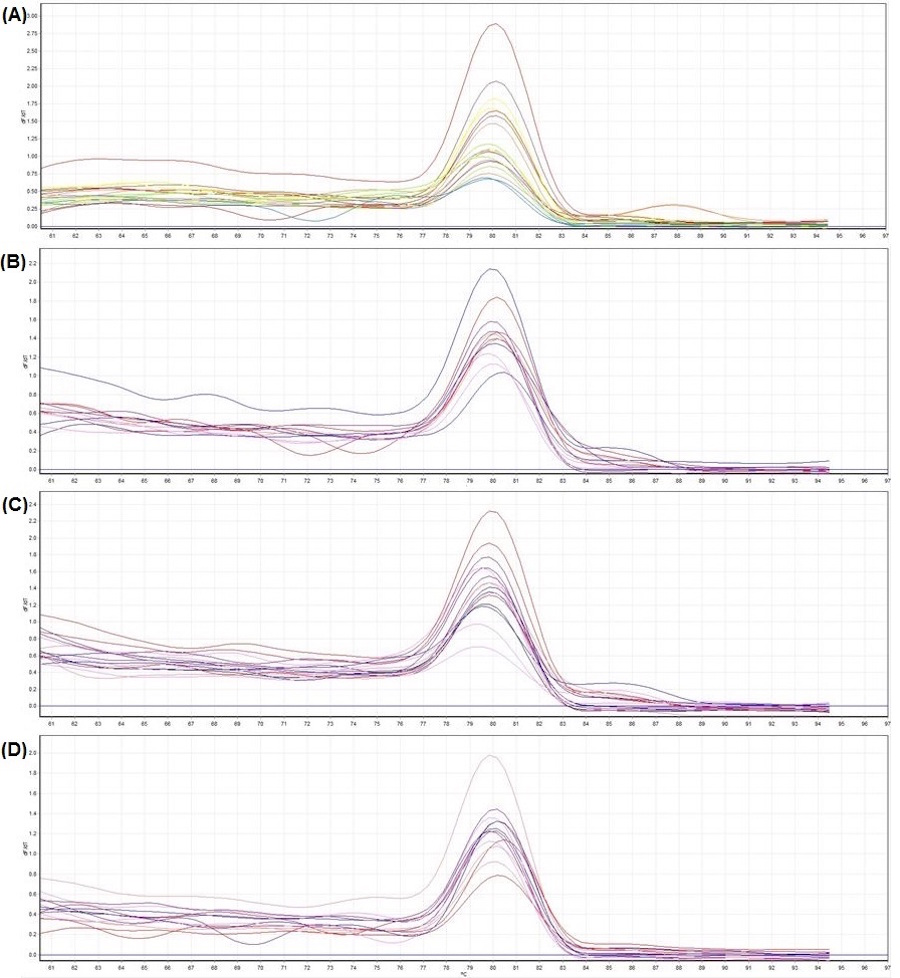

Supplement: Figure S1 — The melting curves for GAPDH expression in (A) liver, (B) lung, (C) small intestine and (D) spleen of red-tailed phascogales (n = 10), performed in triplicate. [file peerj-04-2552-s001.png]

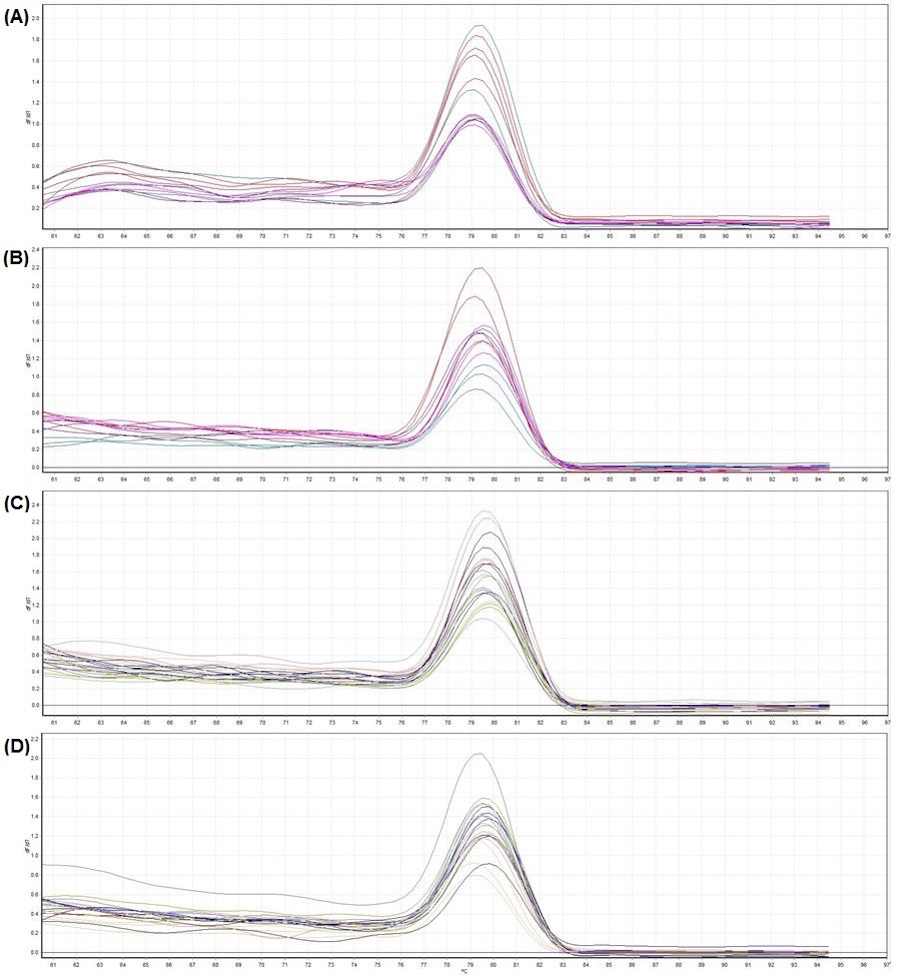

Supplement: Figure S2 — The melting curves for ACTB expression in (A) liver, (B) lung, (C) small intestine and (D) spleen of red-tailed phascogales (n = 10, performed in triplicate. [file peerj-04-2552-s002.png]

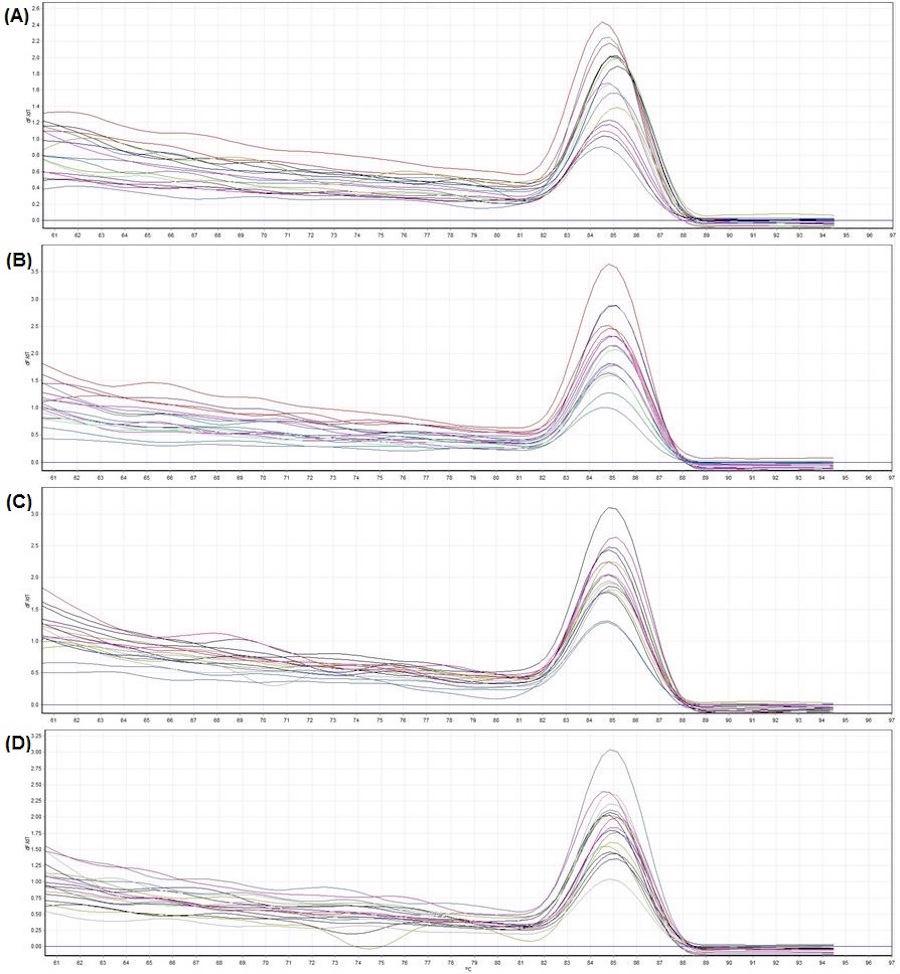

Supplement: Figure S3 — The melting curves for 18S expression in (A) liver, (B) lung, (C) small intestine and (D) spleen of red-tailed phascogales (n = 10), performed in triplicate. [file peerj-04-2552-s003.png]

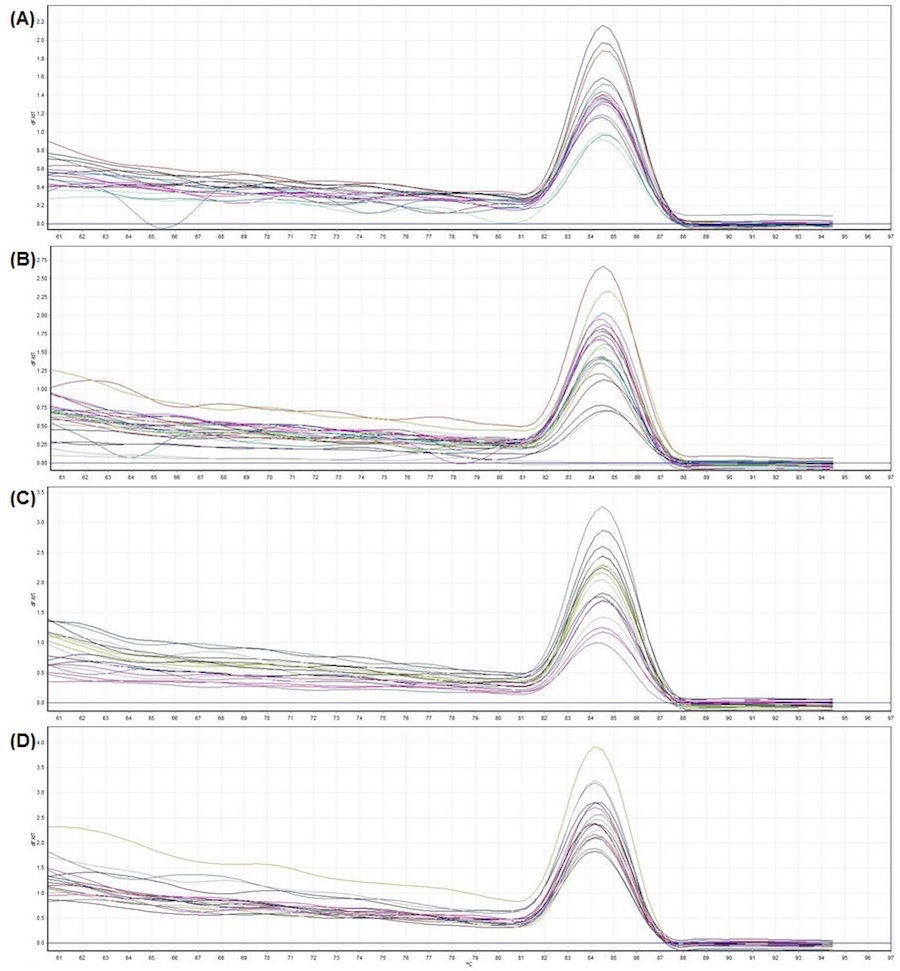

Supplement: Figure S4 — The melting curves for 28S expression in (A) liver, (B) lung, (C) small intestine and (D) spleen of red-tailed phascogales (n = 10), performed in triplicate [file peerj-04-2552-s004.png]

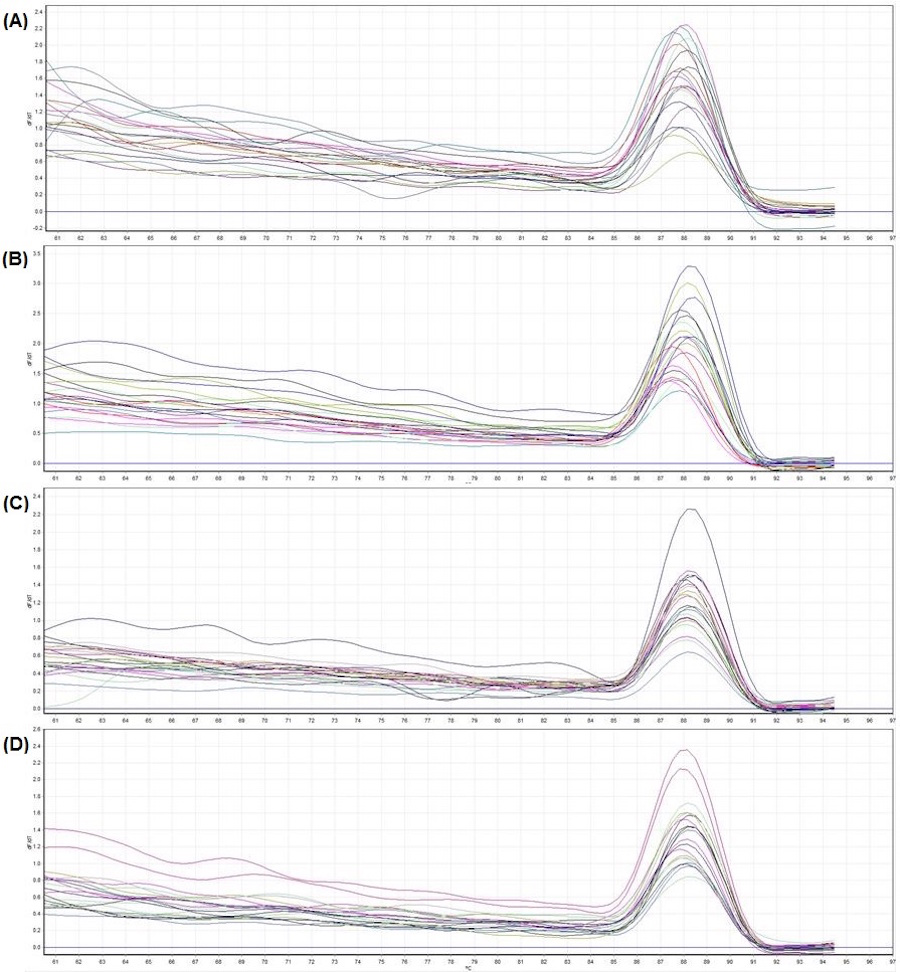

Supplement: Figure S5 — The melting curves for RPL13A expression in (A) liver, (B) lung, (C) small intestine and (D) spleen of red-tailed phascogales (n = 10), performed in triplicate. [file peerj-04-2552-s005.png]
